# Variety of Surgical Guides and Protocols for Bone Reduction Prior to Implant Placement: A Narrative Review

**DOI:** 10.3390/ijerph18052341

**Published:** 2021-02-27

**Authors:** Eitan Mijiritsky, Hadar Ben Zaken, Maayan Shacham, Ihsan Caglar Cinar, Cem Tore, Katalin Nagy, Scott D. Ganz

**Affiliations:** 1 Department of Otolaryngology, Head and Neck and Maxillofacial Surgery, Tel-Aviv Sourasky Medical Center, Sackler Faculty of Medicine, Tel Aviv 6139001, Israel; mijiritsky@bezeqint.net; 2 The Maurice and Gabriela Goldschleger School of Dental Medicine, Tel Aviv University, Tel Aviv 6997801, Israel; 3 Department of Periodontology, Hadassah Faculty of Dental Medicine, Hebrew University, Jerusalem 91905, Israel; 4 School of Social Work, Ariel University, Ariel 40700, Israel; drmaayanshacham@gmail.com; 5 Department of Oral Implantology, Faculty of Dentistry, Istanbul University, Istanbul 34093, Turkey; cinarcaglar@gmail.com (I.C.C.); cem_tore_91@hotmail.com (C.T.); 6 Department of Oral Surgery, Faculty of Dentistry, University of Szeged, Tisza L. krt 64, 6720 Szeged, Hungary; katalin.nagy@universityszeged.com; 7 Department of Restorative Dentistry Rutgers, The State University of New Jersey, Newark, NJ 07103, USA; drganz@drganz.com; 8 Independent Researcher, Fort Lee, NJ 07024, USA

**Keywords:** bone reduction, bone reduction guides, alveolar ridge reduction, ostectomy, surgical techniques guided surgery

## Abstract

Edentulism and terminal dentition are still considered significant problems in the dental field, posing a great challenge for surgical and restorative solutions especially with immediate loading protocols. When the implant placement is planned immediately after extraction with irregular bone topography or there is an un-leveled alveolar ridge topography for any other reason, bone reduction may be required to level the alveolar crest in order to create the desired bone architecture allowing for sufficient bone width for implant placement and to insure adequate inter-arch restorative space. Bone reduction protocols exist in analog and digitally planned methodologies, with or without surgical guides to achieve the desired bone level based upon the desired position of the implants with regard to the restorative outcome. The objective of this paper was to scrutinize the literature regarding the practice of bone reduction in conjunction with implant placement, and to review different types of bone reduction surgical guides. Results: The literature reveals different protocols that provide for bone reduction with a variety of bone reduction methods. The digitally-planned surgical guide based on Cone-Beam computerized tomography (CBCT) scan reconstructed data can improve accuracy, reduce surgical time, and deliver the desired bone level for the implant placement with fewer surgical and restorative complications. The clinician’s choice is based on personal experience, training, and comfort with a specific guide type. Conclusions: Bone reduction, when required, is an indispensable step in the surgical procedure to attain suitable width of bone in anticipation of implant placement ideally determined by the desired tooth position and required restorative space based on material selection for the chosen framework design, i.e., hybrid, monolithic zirconia. Additionally, bone reduction and implant placement can be accomplished in the same surgical procedure, minimizing trauma and the need for two separate interventions.

## 1. Introduction

Edentulism is still considered one of the worldwide most public health significant problems, although improvement in preventive dentistry [1]. According to national epidemiological survey data, even though the rate of edentulous patient is decreasing every decade, the population of elderly people is continuing to grow, as coordinate to the increase in life expectancy; hence, the demand for suitable solution for edentulous patients is growing [1,2].

Patients with a terminal dentition are posing a unique challenge for surgical and restorative solutions, particularly when the treatment plan includes immediate placement of implants after tooth extraction [3,4]. The traditional protocol for terminal dentition was to extract teeth first and then allow sufficient time (in months) for the bone ridge to heal before implant placement would be considered. During the healing phase, the patient received an immediate complete denture until the bone had matured enough for implant placement [3]. Patients would often claim that dentures would impair their quality of life due to pain, denture sores, poor stability, insufficient retention, leading to problems in masticating food properly. In contrast, implant-supported provisional prostheses after immediate loading, remarkably improved the life quality of edentulous patients and were associated with a greater satisfaction of patients in regards to comfort, functionality, phonetics and esthetics [1,5].

As dental implants gained validity and credibility, patients no longer were satisfied with removable dentures and wished for the improved quality of life that implant-supported fixed prostheses can provide [2]. However, there are limitations in that not all patients are good candidates for dental implants, especially those classified as Cawood and Howell Class IV, V, or VI [6]. 

Immediate loading in edentulous patients was introduced more than 20 years ago [7]. Furthermore, long term studies showed that the rate of bone loss was equivalent to the conventional delayed approach therefore acknowledging immediate loading treatment protocols as safe and efficient [8]. As the demand for implant procedures continue to increase, improved and shorter treatment protocols have evolved [9], with certain protocols providing for fewer implants with off-angle placement for immediate surgical and restorative reconstruction [7]. 

Diagnostic imaging technology has evolved with the advent of cone beam computed tomography, interactive treatment planning software applications, computer-aided design and computer-aided manufacturing (CAD/CAM) which provided new digital workflows for enhanced collaboration with dental laboratory technicians for conventional and guided implant placement [3,4]. 

It is undisputed that 3-D imaging modalities and interactive treatment planning software can provide essential information about bone quality, density, and volume for the assessment of potential implant receptor sites at the time of tooth extraction or in healed site [3,4]. The utilization of digital workflows facilitated an understanding of treatment alternatives for terminal dentition or edentulous patients. As diagnosis and treatment planning became more sophisticated, the use of stereolithography (3-D printing) and CAD/CAM surgical guides has expanded from simple pilot drill templates to more sophisticated designs defined as “full-template guidance” [10], digitally planned controlled and accurate bone reduction, sinus-lift guides, harvest guides, and stackable guides directly linking the surgical placement to the restorative outcome [3,4,10,11,12].

The completely edentulous arch presents difficulties during free-hand implant placement due to lack of orientation within the diverse topography which has the potential of mal-positioned implants and proximity or damage to adjacent vital structures. For the partially edentulous patient, there is often lack of symmetry with both soft tissue and bone volume. Hence, the use of 3-D imaging and advanced software applications allows the clinician to detect changes and variations in bone morphology and location of vital structures to accurately determine the favorable treatment plan “prior to the scalpel touching the patient” [9]. 

Advanced 3-D imaging has made it possible to assess bone volume in relation to terminal dentition and irregular bone topography which may remain after tooth extractions. Implant placement can be very challenging without proper diagnostic planning in advance of the surgical intervention. Whenever is possible, while performing bone reduction, care must be taken to remain in the limits of the alveolar bone. The amount of alveolar bone reduction is generally dictated by the creation of a new favorable, functional and aesthetic occlusal plane of the future implant-supported prosthesis. Additionally, complications can be minimized with virtual planning software tools, stereolithography, and CAM-CAM technology [9,13,14].

The purpose of bone reduction is to partly decrease the irregular topography of the knife-edge thin alveolar ridge in the fully or partially edentulous patient in order to create the desired and precise bone architecture that allows sufficient bone height and width for implant placement [3,4,9]. Moreover, the bone should be reduced to establish adequate inter-arch space for the planned prosthesis, referred to as “restorative space” as well as to create a leveled occlusal plane [3,4,8,9,10]. When the patient presents with a high smile line, or a “gummy smile” it may be necessary to recommend bone reduction to improve the aesthetic and functional outcome. When planning the implant-supported fixed prosthesis the smile line must be considered during the planning process to guarantee that the prosthesis tissue junction (PTJ) is not noticeable when the patient’s maximum smile appears, especially in patients with excessive gingival display [15,16,17,18]. This is important due to the complexity related to precise color matching of the prosthetic tissue gingiva with the natural gingival, regardless of the material used. [15,16,17]. The PTJ is relevant to an excessive gingival display in both maxillary or mandibular [16]. 

Often, when planning for extraction and immediate implant placement to achieve the restoratively-driven objectives a certain amount of bone reduction will be required [3,4,8,9,10]. Bone reduction or ostectomy is defined as “the excision of bone or a portion of a bone, usually using a saw or chisel, for the removal of a sequestrum, the correction of a deformity, or any other purpose” [19]. Bone reduction can be accomplished with piezosurgery, or rotary instrumentation. Bone reduction to achieve a flat alveolar crestal bone can be accomplished free-hand, or with a surgical guide generated via CAD/CAM or 3-D printing for the subsequent placement of endosseous dental implants [20]. The main advantage of using a bone reduction surgical guide is that the clinician can virtually pre-plan exactly how much bone needs to be removed in relation to the desired restorative plan, while insuring that remains enough bone height and width for accurate implant placement and stability [9].

The amount of bone reduction required should be determined by the desired occlusion, tooth position, and functional needs. Bone reduction is therefore not arbitrary and should be carefully planned to address the necessary restorative space and reduce surgical and prosthetic complications [4]. Under-reduction of bone can result in poor esthetics due to insufficient room for components to emerge from the implant platform or prosthetic failure as a result of compromised framework design, insufficient restorative space, and potential material fractures. Over-reduction can lead to additional “pink” material in order to make up for an increased vertical dimension of the restoration, necessitate a change in implant lengths and trajectories, and increase the potential risk of injuring vital adjacent anatomic structures [15,16,21]. 

To overcome difficulties in properly estimating how much bone should be removed, the concept of surgical guidance provides an excellent aid that minimizes errors [8,9,15]. Bone reduction templates can be fabricated through a combination of analog and digital methods, i.e., 3-D printing a mandible and fabricating the guide directly on the surface model, or a completely digital workflow via 3-D printing or CAD CAM protocols. Additionally, specific designs require direct anchorage to the bone to achieve three-dimensional stability which is essential to maximizing accuracy [22]. As initially reported, the use of a properly-designed bone reduction guide can also allow for the simultaneous placement of implants in one surgical procedure, greatly reducing treatment time and post-surgical morbidity for the patient. Regardless of technological advances, most bone reduction is still done in a free-hand approach to approximate the required space needed for the restoration, and desired width to place implants [9,13,23]. Pre-surgical prosthetic planning can therefore determine the restorative outcome based on Misch’s Classifications, and how much “pink” will be needed (FP1, FP2, FP3) [24]. 

This present review analyzed currently available literature regarding the practice of bone reduction, and variations in surgical modalities prior to implant placement in patients with edentulous or terminal dentition. 

## 2. Materials and Methods

**Figure 1:**
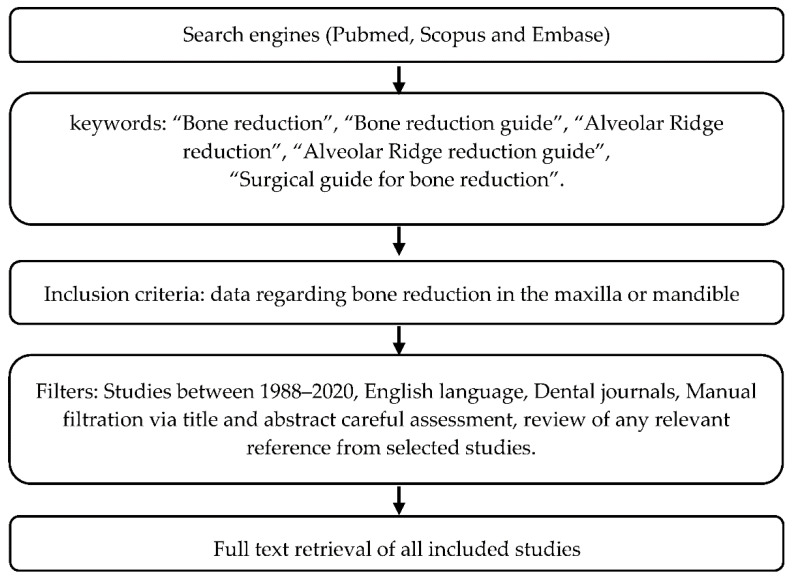
Flowchart depicting the selection process for included studies.

Using the main search engines (Pubmed, Scopus and Embase) a narrative literature review for the subject of bone reduction guides prior to implant placement was performed including papers from 1988 to 2020. The keywords were used in the search engine: “Bone reduction”, “Bone reduction guide”, “Alveolar Ridge reduction”, “Alveolar Ridge reduction guide”, “Surgical guide for bone reduction”. The search was limited to papers in English. Moreover, the literature search was narrowed with respect to dental journals. 116 records were yielded from the search. After articles excluded following title and abstract reading, a full-text file was obtained and assessed for every paper that met the potentially relevant inclusion criteria. The inclusion criteria were articles that included data regarding bone reduction in the maxilla or mandible and variety of surgical guides and protocols for bone reduction prior to implant placement in patients with edentulous or terminal dentition. Subsequently, an additional manual search from the references through reading of selected articles was performed by the authors and completed the list. A total of 42 articles were reviewed (see Figure 1 for graphical flow-chart).

## 3. Results

### 3.1. Bone Reduction Guides: Techniques and Protocols

When applicable, bone reduction is essential to level the bone to gain adequate width for implant placement with enough vertical restorative space to fabricate the prosthetic reconstruction at the correct vertical dimension of occlusion (VDO) [25,26,27]. Bone reduction often removes many of the anatomical landmarks that clinicians depend on with free-hand implant placement, and therefore represent a challenge to place implants in the planned position while avoiding harm to adjacent vital structures [4,9]. Hence, implant surgical drill guides, and now bone reduction guides have become more popular due to advances in 3-D imaging and digital fabrication technologies. There are various techniques and protocols that have evolved to provide adequate bone reduction for implant reconstruction as described here within. The following subsections entails various bone reduction guides, along with indicated techniques and protocols (see Table 1 for summarization of included studies).

#### 3.1.1. NOVUM System

In 1999, the NOVUM system was introduced by Braånemark and colleagues [7]. This surgical and prosthetic protocol allowed for the immediate loading of three wide implants with a definitive fixed prosthesis delivered on the day of the surgery. An essential foundation for pre-surgical planning was to have accurately mounted casts at the proper vertical dimension of occlusion which recorded the inter-arch relationship. Using a lateral cephalometric radiographic image, the clinician would determine the most desirable implant position based on the volume and morphology of the existing bone. The implant was positioned within the residual ridge to a depth that would provide the required width of bone (at least one millimeter facial and lingual) utilizing a clear overly from NOVUM. The dental laboratory technician would then translate the radiographic position to the working cast, manually reduce the stone to then fabricate a bone reduction template which fit over both maxillary and mandibular arches for stabilization made from a transparent acrylic resin. The template would then be seated over the exposed bone, containing a window to allow access to surgically remove and then flatten the alveolar crestal bone. The buccal and lingual arm extensions surrounding the window of the bone reduction template were reinforced by metallic wires. The template held the upper and lower jaw open allowing clear visibility and access during the surgical procedure. While simple and cost-effective, the accuracy of the bone reduction was limited by the analog methods utilized, and inaccuracies in determining the plane of occlusion which could result in a tilted occlusal table [22].

#### 3.1.2. “All-On-Four” Concept

The protocol as described by Maló was to place four implants for both the mandible and the maxilla to support an immediate-load full-arch all-acrylic prosthesis. The procedure was accomplished for either a dentate or edentulous arch with the posterior-most implants placed in a “tilted” fashion to gain increased anterior–posterior spread while avoiding vital anatomical structures. To achieve consistency and accuracy a specially designed flat metal guide was utilized, indicating the position of the implants within the envelope of the occlusion. The guide was stabilized with a short 2 mm osteotomy at the midline of the jaw and the titanium metal guide was “bent” to accommodate accurate positioning of the implants within the desired occlusal relationship.

When teeth were present, they were all extracted, flaps were raised to expose the alveolus followed by free-hand leveling of the bone to provide a flat plane so that the metal guide could be utilized. For an edentulous arch, a similar leveling of the bone was achieved after the alveolus was exposed. While these steps were not explicitly stated within the articles, the photographs revealed that the bone was reduced as an integral part of the protocol. A fully guided protocol was subsequently described for a flapless surgical procedure in completely edentulous patients aided by computer software and surgical hardware. Bone reduction was not part of this protocol for immediate loading on four implants [28,29].

#### 3.1.3. Guided Bone Reduction and Drill Template 

**Figure 2:**
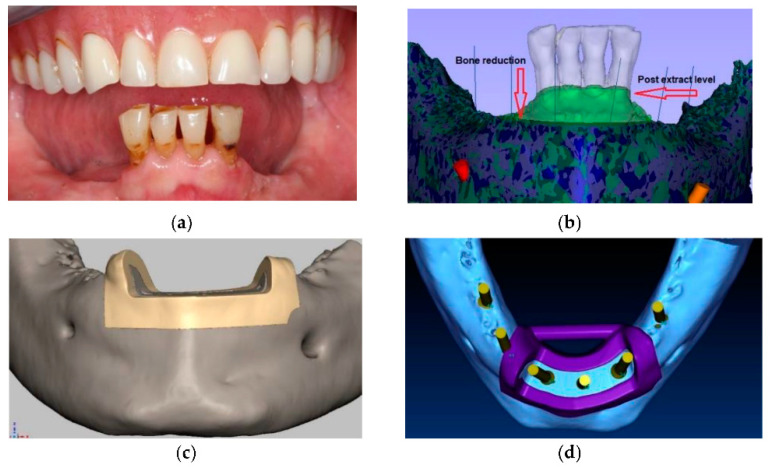

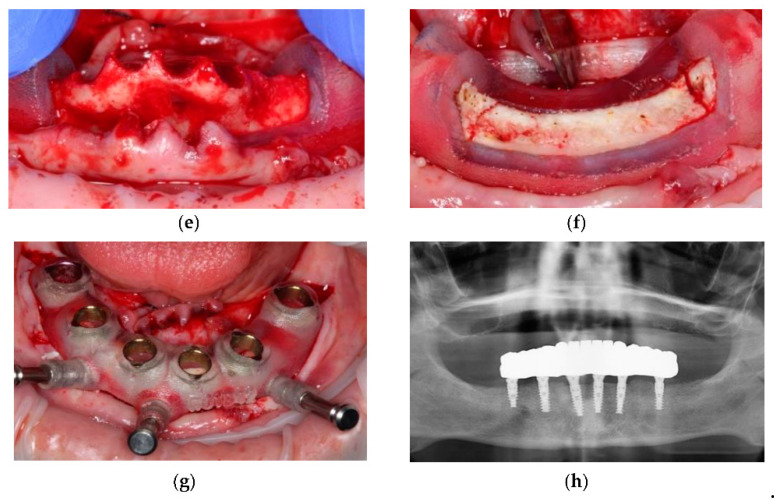
Example of a case using bone reduction guide: (**a**) Pretreatment clinical situation. Frontal view of mandibular arch; (**b**) Scans of the initial clinical situation of the patient. Horizontal arrow shows the bone level after extraction, vertical arrow shows the desired bone level after reduction; (**c**) Computerized virtual mandibular alveolar bone reduction surgical guide and the bone level after reduction; (**d**) Computerized virtual for implant position after bone reduction; (**e**) Acrylic resin bone reduction surgical guide in position after tooth extractions, prior to bone reduction; (**f**) Alveolar ridge reduction according to virtual plan and surgical guide; (**g**) Second surgical guide used to insert dental implants according to pretreatment virtual plan. Anchoring pins secured guide in the right position after bone reduction; (**h**) Post-operative panoramic radiograph of implants and definitive prosthesis.

When the patient exhibits loss of alveolar bony architecture post tooth loss, it often results in an extremely narrow, thin, and fragile anterior segment of the mandible or maxilla. Due to the limitation of buccal-lingual dimensions, implant placement can be compromised. In many cases bone grafting may be necessary to add necessary width for implant placement. However, if there is adequate bone height and width present in the remainder of the alveolus, surgical reduction of the thin residual ridge can allow for suitable width to accommodate implant placement and initial stability. To achieve the desired amount of bone reduction a novel concept was developed in 2002 and first published in 2006 illustrated pre-surgical virtual planning steps combined with the use of 3-D printed stereolithographic models to improve accuracy of both bone reduction (mandibular edentulous ostectomy) followed by guided implant osteotomies [9]. Separate sequential 3-D printed templates were fabricated to facilitate the procedure. It should be noted that the virtual planning of the implants based on restorative outcomes was the defining step to determine the amount of bone reduction required. Using the three-dimensional dataset and interactive treatment planning software the position of the implants were determined first, and then a 3-D printed bone reduction template was fabricated to sit on top of the bone, allowing access for bone reduction. After sufficient full thickness mucoperiosteal flap exposed the thin alveolar ridge, the first surgical template was seated on the exposed mandibular symphysis with an occlusal window to provide clear visualization and guidance as to the amount of bone reduction required of the knife-edged bony ridge. Due to stability on the residual bony ridge, there was no need for fixation or anchor pins as shown in Figure 2A–E. Once the ostectomy was completed and the bone flattened, sequenced bone-borne drill guides were then seated over the newly flattened bone to accurately produce osteotomies with sequential drilling protocols for dental implant placement, each to carefully expand the osteotomy based on manufacturer-specific diameters to ensure correct delivery of the implants as shown in Figure 2F–H.

The templates controlled depth, trajectory, and diameter to achieve accurate placement of the implants within the bone [1,9,23]. 

#### 3.1.4. A Technique for Transferring a Patient’s Smile Line Using Gutta-Percha

While CBCT and interactive treatment planning software can provide clinicians with excellent information to assess the individual patient’s anatomical presentation, there are many steps that can increase the value of the scan. This would include the “scannographic” template, which was developed to indicate the desired tooth position using a radiopaque material incorporated into an appliance usually a duplicate of the patient’s denture or diagnostic wax-up fabricated in advance, and then worn during the image acquisition. By using this method, the clinician can easily determine the desired tooth position versus the underlying bone [30,31]. However, it is often difficult to incorporate the aesthetic values of the information from a radiopaque guide as it relates to patients that exhibit excessive gingival display. To enhance the diagnostic quality, prior to the scan acquisition, a clear radiographic “smile guide” was produced using radiopaque gutta-percha markers to indicate the most apical position of the upper lip as elevated during a smile. Additional posterior oblique markers were also incorporated as fiducial markers to aid in the planning of implants. The CBCT data was transferred to an implant planning software program (Invivo 5; Anatomage, Inc. Santa Clara, CA 95054-3105, USA for analysis. The ability to visualize the radiopaque gutta percha helped to quantify the amount of bone reduction needed and the most favorable position of the implants to ensure sufficient prosthetic space if there is enough remaining bone volume [16,17]. During surgery, before extracting teeth, a full-thickness mucoperiosteum flap raised and the radiographic smile guide for denture with terminal dentition (for edentulous patients a clear duplicate denture is fabricated) seated on the teeth. Using a sharp sterilized graphite wooden pencil, a line right above the gutta-percha marker was drawn. After the guide was removed, the bone was then reduced at least 4 mm below the pencil mark. Another clear duplicate denture placed as a surgical template to visualize and confirm if sufficient bone was reduced. Subsequently, the implants were placed as decided in the treatment plan [16]. 

#### 3.1.5. Stackable Guides

As technology has improved, the linking of the various components became a digital reality. Therefore, it became possible to plan for the bone reduction, the drill guide, and the link to the restoration of the day of surgery. To achieve success, it is essential that all patient records are obtained which include the CBCT scan, intra-oral scanners (IOS) or physical impression of the existing dentition, intra and extra-oral photographs, diagnostic wax-ups, and a determination of the proper vertical dimension of occlusion. A CBCT is essential for accurate implant planning achieved by evaluating all views, including the volumetric reconstruction. Due to the inherent limitation of the CBCT volumetric reconstruction, the virtual surface model does not represent true surface detail. Therefore, the digitization of the intra-oral condition alone or with a diagnostic wax-up can be achieved with either intra-oral optical scan or desktop scanner to be then merged to the CBCT scan to provide the most accurate method for diagnosis and treatment planning. Using all of this information the dental laboratory can design and fabricate the transitional prosthesis and all components for the surgical intervention in advance. 

A “stackable guide” protocol starts by confirming the original occlusal bite registration with a clear splint made to validate the bite prior to administering anesthesia. Once a full flap was reflected, the remaining teeth were extracted. Then, the precise location of the first guide, a “bone foundation guide” for bone reduction was validated by bite index to the opposite arch. Once the guide position was verified, it must be fixed to the alveolus with anchor pins. The guide struts are then removed to allow reduce the bone to the level of the guide. After the bone reduction procedure is complete, the next guide for implant placement is pin-indexed onto the first guide. Then, following the proper surgical drilling sequence, the implants are placed with depth and rotational control as delivered through the template with manufacturer-specific carriers defined as “full template guidance” [10]. The prosthetic abutment components can then be delivered to the implant. The implant mounts and the surgical drill guide, the transitional prosthesis can then be placed onto the multi-unit abutments using a silicone spacer which is placed on the bone foundation guide to locate the prosthesis at the precise centric relationship and vertical and to reserve the previous tissue height. Then the occlusal registration is done while the patient bites into the clear splint [1]. The titanium sleeves can then be secured to the prosthesis with a dual-cure restorative material, removed, polished and cleaned. Upon delivery to the patient, the implants can then be immediately loaded with the use of the stackable guide system [1]. 

A similar system has been developed to produce metal stackable guides that attach only the buccal surface of the bone. Using a “pin-guide” the fixation-base is anchored to the bone over the existing teeth. The pin-guide is then removed, and the fixation-base remains to serve as the bone reduction guide. Once the amount of bone reduction is confirmed, a drill guide is attached to the fixation base, and all implant osteotomies are completed. The drill guide is then removed and replaced with a “carrier” guide which has posts to orient the transitional prosthesis over the multi-unit abutments (MUAs). Using dual-cure material, the titanium sleeves are luted to the prosthesis. The prosthesis can then be removed, any voids are filled, polished, and delivered to the patient after soft-tissue closure with sutures [11,32,33]. 

#### 3.1.6. Guides for Patients with a Terminal Dentition

Surgical drill guides can be tooth borne, bone borne, or mucosal borne. However, when tooth extraction followed by bone reduction and immediate implant placement is planned, more than one template is often required. The templates can be fabricated via 3-D printing and SLA stereolithography, CAD CAM, or laser-metal sintering. To improve accuracy, the templates should be registered and stabilized to the hard tissue anatomy, often accomplished with fixation or anchor pins. A bone reduction concept was described by Alzoubi et al. (2016) [4] utilizing multiple CAD CAM templates registered to the mandibular bone with fixation pins guided by the terminal dentition. The ‘‘3-guide technique” includes 3 CAD/CAM surgical guides. The first guide utilizes the existing terminal dentition to position and drill the fixation/anchor pins to the bone. This technique can obviate problems associated with unstable guides that may be difficult to stabilize after tooth extraction by using anchor pins to minimize movement of the guide during surgery. Each guide was fixated with three anchor pins in the same three anchor holes to provide the same registered position for all subsequent guides. The second template was a bone-supported surgical guide that seated over the teeth and onto the surrounding bone, fixated with the same anchor pins in the same position as the initial guide. The teeth could be extracted before or after the second template, followed by bone reduction to the adequate bone level as indicated by the second guide. The final guide was then fixated with the anchor pins, providing the drilling platform for implant placement. The implants were placed using a fully guided implant kit [4].

#### 3.1.7. The 3A-2B Rule

The 3A-2B rule was utilized to establish bone and soft tissue contours for an implant-fixed prosthesis (FP1) “All-on-Four” prosthetic design [21]. According to the desired tooth design from a computer simulation and using used DSD (digital smile design) for planning single tooth to complete arch implant-supported rehabilitations for an edentulous patient [3]. Originally described for partially edentulous presentations, it was possible to fabricate a temporary prosthetic according to the desired optimal teeth length and ovate pontics to maximize aesthetic outcomes and the temporary prosthetic was produced and used as a surgical guide. In this protocol, using the margin of the temporary prosthetic as a guidance, the bone reduction was accomplished by measuring the distance from the bone to the margin of the cervical contour planned crowns was less than 3 mm the bone was reduced in a free-hand method to ensure a 3 mm space for suitable biological width thickness. Furthermore, the temporary prosthetic used also as a guide for implant placement [21]. Following 3A-2B rule, a concept that was originally create for single implant placement in 2013 [34] and then modified in 2017 for complete edentulous [21], implants were placed 3 mm apical away from the crowns’ margin to create a gap for biological width and to avoid buccal bone resorption, the implant positioned 2 mm from the buccal bone. It should be noted that these parameters may not apply universally to all implant systems with differing platforms and restorative connections. For his protocol, a complete denture as modified to prevent any contact with the healing abutments. 12 weeks later, after the tissues recovered, the full-arch zirconia prosthesis was placed and the patient occluded for 20 min as to fit the temporary prosthesis to the abutments. Two weeks post-insertion, the soft tissue adapted to the prosthesis shape, providing the desired new gingival esthetics contour [21].

#### 3.1.8. GuidedSmile Chrome-The “Scalloped Guide”

This stackable guide system was developed to manage an FP1 prosthetic design usually in the maxillary arch, with the goal to preserve bone interproximally after tooth extraction. Using the GuidedSmile Chrome metal-guide concept, and also developed separately in resin, the bone is not reduced horizontally, it is reduced based on tooth morphology using a “Scalloped Guide” [20]. The pre-surgical evaluation will confirm a terminal dentition with intact alveolus and minimum bone resorption (Cawood and Howell Class I, II, and III). This technique provides static guides fabricated via CAD/CAM, 3-D printing, or laser metal sintering to first “scallop” the bone after extraction, leaving an intact interproximal bone morphology in a less aggressive reduction therefore when possible avoiding flattening of the alveolar crest. The guide design is predicated on excellent pre-surgical prosthetic planning. This includes an understanding of the occlusion, inter-arch space, bone volume, density, and morphology. Once the pretreatment scan and wax-up scan merged with the CBCT images, the osseous recontouring is designed and simulated on the computer to establish the ideal restoration’s contours. Using the desired emergence profile of the restoration the trajectory and depth of each implant is confirmed to within the envelope of the teeth, a scalloped bone reduction guide is designed to achieve preservation of the interproximal height of bone for an FP1 full arch prosthesis. Using either a stackable type guide or an interchangeable set of guides, the teeth will be extracted, followed by the bone recontouring, and then a separate drill guide to create the osteotomies and place the implants. The pre-fabricated transitional restoration will then be positioned onto the guide and luted to titanium sleeves attached to the abutments. The temporary screw-retained poly(methyl methacrylate) (PMMA) is designed to allow the suitable natural emergence profile for the immediately loaded implant supported teeth and pontic areas. Closure will be achieved after grafting to fill any remaining extraction sockets, defects, or concavities to support the soft tissue. The ideal healing period is approximately 3-4 months to allow for soft and hard tissue maturation prior to delivery of the definitive screw-retained monolithic zirconia restoration [20,35].

#### 3.1.9. Magnetically Connected Guides

This stackable guide system utilizes magnets as a means to connect different surgical guides. Using a fully digital workflow includes facial scan, DSD, IOS, CBCT M and more, guided alveolar ridge reduction was fabricated. The surgical guide was fixed with anchor pins to direct the high and depth of the bone reduction. The guides contain 3 mm diameter magnet discs that fit tightly in the attachments and enable accurate fit and stability of the bone reduction guide to the next guide, the implant surgical guide. After the implant placement is done, the implant surgical guide is detached, screw-retained abutments are connected to the implants and the interim temporary PMMA prosthesis is magnetically attached to the bone reduction guide in order to capture the screw-retained abutments with bis-acryl composite resin. Lastly, the abutments are unscrewed and the temporary prosthesis is detached so the bone reduction guide can be removed and an immediate temporary implant-supported prosthesis is finally adjusted and installed. One disadvantage of this guide is that due to the use of magnets there is slightly increased cost of treatment [36].

## 4. Discussion

Manual, analog bone reduction techniques were documented for ideal implant placement with the Brånemark Novum implant system, more than 20 years ago, originally utilized for immediate loading of patients with mandibular edentulism. In this protocol, the bone reduction was accomplished in the anterior mandibular symphysis region to gain sufficient bone width to accommodate implant placement with a free-hand protocol using twist reamer drills with sterile, isotonic saline solution. The height of the alveolar crest was reduced to achieve approximately 7 mm bone width for the surgical implants guide templates [7,37].

In edentulous patients, the presence of undercuts or prominences in the bone can compromise the accuracy of the desired prosthesis by changing the maxilla–mandibular relationship and proper arrangement of artificial teeth [38]. The bone reduction procedure has become an established treatment option and an indispensable step in the surgical procedure to attain suitable height and width of bone for implant placement where a knife-edge alveolar ridge is present and increase available inter-arch space for adequate restoration. Furthermore, the procedure is done to increase restorative space without compromise of phonetics, esthetics or appropriate vertical occlusal dimension (VDO) [23,38,39,40]. 

Especially in edentulous patients, it is essential to make an accurate preoperative treatment plan and diagnosis to attain predictable results and satisfying clinical outcomes. [15,38]. When reducing the bone high prior to prosthetic treatment it is important to reduce the minimum amount as the bone is invaluable for implant placement and denture support [38].

Current trends endorse a surgical guide approach for the procedure of bone re-contouring, reducing bone abnormalities that interferes with prosthetic rehabilitation [38]. In order to minimize complications, reduce surgical time, and improve accuracy in the bone reduction process, several innovative pre-surgical prosthetic diagnostics tools can be employed. Depending upon the patient presentation, i.e., dentate or edentulous, certain steps may be required prior to acquiring a three-dimensional scan. The CBCT scan data can be translated into a treatment plan for esthetic, functional and predictable results by fabricating an accurate surgical customized guide prior to the time of surgery [9,23]. To achieve true restoratively-driven implant placement, the prosthetic outcome must be approved and designed in advance. The surgical guide is the connection between the treatment plan and understanding the patient anatomy to the execution of the desired restorative outcomes. With an edentate patient, it is essential to have a diagnostic wax-up or virtual tooth set-up at the proper centric and vertical dimension of occlusion that will aid in the strategic placement of dental implants. Prior to the necessary scan, fiducial markers should be used as an aid to the superimposition or merging of the datasets to ensure accuracy. This step may not be needed for a dentate patient because the teeth themselves can serve as reference points. Therefore, the foundation for proper planning will be determined with the acquisition of the CBCT scan to assess the individual patient’s bone volume, density, quality, pathology, and adjacent vital structures in relationship to the desired position of the restoration. Implants will then be planned based on the restorative space necessary and the need for bone reduction resulting in the fabrication of surgical guides through stereolithography (3-D printing) or CAD CAM protocols [9,20,23,41] Pre-surgical prosthetic planning can therefore determine the restorative outcome based on Misch’s Classifications, and how much “pink” will be needed (FP1, FP2, FP3) [24]. 

A surgical guide is significantly important for the evaluation of the amount of bone needed to reduce during the surgical procedure, particularly when a large amount of bone must be reduced [13,38]. The surgical guide has been proven to be more precise and safer, with greater efficiency and predictable results than the traditional free-hand of bone reduction and implant placement [4,9]. By taking out the guesswork of the equation as in free-hand and instead based the treatment plan on the bone anatomy, vital anatomical structure and restorative needs as in surgical guides, successful and better treatment outcome can be achieved [9].

It is easier and simpler for the clinician to reduce irregular bone precisely according to the surgical guide and forming the preferred bone level when all of the decision-making and treatment planning was done in advance [9]. There are diverse types of surgical guides available; the choice of the clinician is based on his preferences and convenience with a particular guide as a suitable for the patient’s treatment plan [38]. 

The advantages of using surgical guides CT-derived for bone reduction are as follows: improved accuracy, reduced surgical time, easer to perform a successful immediate loading procedure at the desired position of the implants as planned in advance and with fewer surgical and restorative complications [9,23]. Possible drawbacks of the bone reduction procedure can be: extensive reduction of the residual ridge, excess mobilized keratinized mucosa and loss of cortical bone [39,42].

During mechanical stress as part of bone remodeling and implant insertion, oral-derived stem cells may respond to such activity via mechanosensitive mechanisms, and respond along with different cytokines and growth factors. Such mechanisms are suggested to play a vital role in bone remodeling processes [43,44].

The study has several limitations including its narrative approach. This approach was decided due to the variance across the different techniques, with paucity of high-quality studies for each technique. Future studies regarding such techniques should include higher quality studies, e.g., randomized controlled trials. In addition, the current review did not address the management of surgery and its impact on bone remodeling. For such cases, we refer the reader to relevant studies [45]. The impact of bone reduction in patients with systemic alterations was also not addressed in the current review [46]. When coming to perform an implant supported rehabilitation in cases of terminal dentition or full-arch edentulism, it is important to perform the treatment in the safest and fastest way. Therefore, in those cases which require bone reduction, a bone reduction guide has been proven to be very effective and safe tool during the treatment [15].

## 5. Conclusions

It can be concluded that ever since implant therapy became a common procedure in patients with terminal dentition or edentulism, bone reduction procedures became an established treatment option and an indispensable step in the surgical procedure to attain suitable bone level for implant placement and increased available inter-arch space for adequate restoration. In current protocols based on advanced digital workflows, the bone reduction procedure is commonly performed with a surgical guide (usually CAD/CAM generated) that helps to achieve more accurate, efficient, safe and predictable results and reduces surgical time. Because the ability to use digital workflows for a treatment plan is now within the reach of every clinician, bone reduction guides are very familiar; and therefore, there is a wide variety of bone reduction guides in the market. This review lists the existing methods in the market and their clinical applications. The practicing clinician should be familiarized with important modulators such as oral-derived stem cells because these may exert their effect on bone reduction and implant insertion procedures. The choice of the clinician is based on his preferences and convenience with a particular guide as suitable for the patient’s treatment plan. 

## Figures and Tables

**Table 1:** Summary of included studies.

Method	Study Design	Perform Bone Remodeling in Full-Arch Restorations (Mandible or Maxilla)	How to Estimate the Amount before Surgery?	What Is the Rationale for Each Technique	Clinical Outcome
NOVUM System [7,22]	50 participants (mandible), 150 implants, 6 mo-3 y follow-up [7]. 20 patients (mandible, 10 with stents, 10 without) [22]	Yes	Panoramic, lateral and intraoral periapical radiographs [7], Lateral cephalometric [22]	Introduction of one-stage implant insertion and prosthetic rehabilitation [7]. Surgical stent fabrication to support the NOVUM system [22].	98% survival rate, 98% prosthetic survival rate. Increased patient satisfaction; −1.25 mm mean marginal bone loss across 3 y follow-up [7]; Simple and cost-effective; Potential inaccurate plane of occlusion leading to tilted occlusal table. Increased prosthetic predictability (*r* = 0.9215) for patients treated using a stent [22].
“All-on-Four” Concept [25,26,27,28,29]	Ref [25,26,27]—reviews 23 patients (both arches), 92 implants, 6 mo-21 mo follow-up [28] 111 patients (both arches), retrospective study of 532 implants (between 2005–2010) [29]	Yes	Clinical, Surgical software and radiographs (panoramic, CBCT).	Helps to avoid nerve injury, select implant sites, and establish the biomechanical advantage of increased anterior-posterior axis [27]. Nonaxial implant insertion and reduced number of implants [25].	97.8% survival rates, mean marginal bone loss 1.9 mm [28]. 94.5% survival rates, 97.8% prosthetic survival rates, mean marginal bone loss 1.3 mm, bruxing and smoking had negative effect on implant outcomes [29].
Guided Bone Reduction and Drill Template [1,9,23]	Ref [1,9,23]—reviews	Yes	Ref [1]—Using CBCT data, clinical photographs, full-arch impressions and bite registrations [1] Ref [9]—CBCT and stereolithographic model fabrication [9] Ref [23]—CBCT and innovative softwares, followed by mucosal-supported template fabrication using 3D printing, stereolithographapy, or CAD-CAM process [23].	Increased precision and accuracy of surgical and immediate provisionalization [1]. In cases of irregular bony architecture; inability to accurately determine relationship between desired tooth position and underlying bone [9]. Better understating of patient’s individual anatomy and proximity of vital structures, eliminating usage of ancillary appliances [23].	Less time consuming procedure, increased patient satisfaction [1]. Predictable immediate prosthetic outcome; Increased accuracy using immediate insertion of parallel implants [9]. Reduced surgical and prosthetic complications, reduced laboratory work, better understanding of relationship between prosthesis and underlying structures [23].
A technique for transferring a patient’s smile line using gutta-percha [16,17,30,31]	Ref [16,17]—Technical study	Yes	Clinical evaluation and CBCT.	Accurate bony reduction, especially in cases of limited prosthetic space, excessive mandibular gingival display, and esthetic zones.	Highly accurate and esthetic
Stackable Guides [10,11,32,33]	Case report [11]	Yes	CBCT and computer software	Accurate reduction using specialized software utilising CAD/CAM and 3D-Dicom data	
Guides for Patients with a Terminal Dentition [4]	Retrospective study. 5 patients (both arches), 26 implants	Yes	CBCT and computer software	Utilizing the terminal dentition to increase guide stability	Overall deviation means—1.98 mm
The 3A-2B rule [3,21,34]	Clinical report with 3y follow-up [21]. Clinical report with 1y follow-up [34]	Yes	Clinical photography, computer software (DSD, Radiographic Biological Ruler © [34]), CBCT, CAD/CAM [21]	Increased esthetics	Re-established maxillary incisal curve and lower lip relationship [21], Optimal restoration design [34]
GuidedSmile Chrome- The “Scalloped Guide” [20,35]	Technical study [20]	Yes	Clinical photography, computer software (DSD), CBCT, CAD/CAM	Minimize bony reduction and obtain accurate reductions; aids in hide the “transition zone”, optimize cleans ability	
Magnetically Connected Guides [36]	Technical study	Yes	Intra-oral scanner, clinical photographs, CBCT, CAM/CAM	Improved stability utilizing the magnets-embedded PMMA prosthesis	-

CBCT (Cone Beam Computerized Tomography); CAM/CAM (computer-aided design and computer-aided manufacturing); DSD (Digital Smile Design); PMMA (Poly(methyl methacrylate).

## Data Availability

Not applicable.
